# Deep learning enhancing banking services: a hybrid transaction classification and cash flow prediction approach

**DOI:** 10.1186/s40537-022-00651-x

**Published:** 2022-10-02

**Authors:** Dimitrios Kotios, Georgios Makridis, Georgios Fatouros, Dimosthenis Kyriazis

**Affiliations:** 1 Department of Business Administration, University of Piraeus, Karaoli ke Dimitriou 80, Attica, 18534 Athens, Greece; 2 Department of Digital Systems, University of Piraeus, Karaoli ke Dimitriou 80, Attica, 18534 Athens, Greece

**Keywords:** Data analytics, Deep learning, Time series forecasting, Transaction categorization, Surrogate data, Cash flow prediction

## Abstract

Small Medium Enterprises (SMEs) are vital to the global economy and all societies. However, they face a complex and challenging environment, as in most sectors they are lagging behind in their digital transformation. Banks, retaining a variety of data of their SME customers to perform their main activities, could offer a solution by leveraging all available data to provide a Business Financial Management (BFM) toolkit to their customers, providing value added services on top of their core business. In this direction, this paper revolves around the development of a smart, highly personalized hybrid transaction categorization model, interconnected with a cash flow prediction model based on Recurrent Neural Networks (RNNs). As the classification of transactions is of great significance, this research is extended towards explainable AI, where LIME and SHAP frameworks are utilized to interpret and illustrate the ML classification results. Our approach shows promising results on a real-world banking use case and acts as the foundation for the development of further BFM banking microservices, such as transaction fraud detection and budget monitoring.

## Introduction

Small Medium Enterprises (SMEs) are the backbone of the global economy as, according to Organisation for Economic Cooperation and Development (OECD) [1], SMEs account for 99% of all enterprises and over 60% of value added in its member nations. In Europe, SMEs play an important role, accounting for 99.8% of all firms in the EU-28 non-financial business sector (NFBS), while creating 56% of value added and driving employment with 66% [2]. SMEs worldwide face a complex and challenging environment due to swift global business changes and the ongoing digital revolution. The on-going covid-19 pandemic widens SME’s liquidity gap, but is also heavily affecting customer behaviors and market transactions through higher supply chain integration and increased product differentiation. The adoption of digital technologies and cutting-edge tools can empower SMEs by lowering operational and business costs, saving time and valuable resources, particularly for firms that exhibit a slowdown in economic activity and smaller volumes of production, as well as having limited market reach and bargaining power with stakeholders [3].

Radical technology advancements may have an impact on nearly every area of a SME’s operation, resulting in data-driven strategic decision-making procedures. An SME’s digital transformation could provide a fresh perspective on business and financial management, resulting in a competitive advantage through increased productivity and quality control, the introduction of new marketing techniques, and the ability to identify new markets and anticipate business opportunities.

However, the digital transformation of an SME hinders many risks and challenges, having to do both with accessing the business’s data, as well as analyzing them. According to the 2019 OECD SME Outlook [1], the main barriers to SMEs digitalization are the limited digital skills found in most SMEs management team and strict data protection regulations. Of course, not all industries confront the same issues and hurdles to digital transformation. The financial sector appears to generate more data and use data analytics more frequently [4], with some innovative start-ups and fintechs paving the way for the rest of the SMEs struggling to participate in the data-driven economy.

It is also noted that some commercial data analytics applications are starting to be available horizontally for SMEs through cloud computing services offered by large corporations, allowing SMEs to access tailored A.I. services even when they lack the internal resources to develop them [5]. Other commercialized applications are included in ERP or Accounting Software packages. Stand-alone BFM tools and frameworks are also available for commercial use, but most of them are geared towards analyzing only past transactions, making such tools inadequate in today’s world.

Banks, retain data of SME customers as required for their main services. .This could offer a business financial management toolkit to their customers by leveraging all available data , providing value added services on top of their core business. In that direction, banks can harness all available data to provide accurate business insights and analytical services to retail or business customers resulting, as noted by Winig [6], to increased customer base and engagement.

However, developing personalized segmented services is not a straightforward task for banks, as it poses a variety of business and technical challenges. From a technical perspective, a key challenge that arises during the development of data analytics services and the utilization of supervised ML techniques for the financial sector, is the lack of labelled data and the frequent appearance of non-numeric data. Considering the volume of financial data generated or utilized by banks, hand labelling the data becomes a cumbersome task and developing personalized services could prove both resource demanding and time consuming. Moreover, most of today’s Financial Management tools for Small Medium Enterprises (SMEs) are geared towards analysing only past transactions, utilizing mainly statistical methods, making such tools inadequate in today’s world. Today, SMEs and their customers alike, demand just-in-time processing, transparency and personalized services to assist SME owners not only in understanding better their SME business/financial health but also to be able to model their options and to decide on the next best action to take. Most prevalent techniques for financial data extraction include rule-based approaches and supervised machine learning methods aiming at text extraction. When rule-based approaches are followed, keeping all the rules up to date and regularly updating them due to new customers, new scenarios or changing consumer behaviors, in many cases is a real headache for practitioners.

This paper addresses the emerging challenge of SMEs financial management by introducing a data-driven approach to facilitate timely decision making for SMEs. In this context, the operational objective is twofold:

1. Timely transactions categorization based on efficient classification of each transaction according to the underlying product/service. Providing a sound approach for how multidimensional tabular data along with unstructured text data (i.e. transaction description) can be classified using AI based methods and represented in the time domain as time-series enabling the exploit of the forecasting method.
2. Systematically forecast inflows and outflows based on historical data applied for each general transaction category, by applying and comparing different probabilistic Deep Learning. This is extended towards the utility of surrogate data as a dataset enrichment approach.

While the scientific contributions that support and extend the proposed solution can be briefly summarised in terms of added value, as the proposed approach enables:

1. Continuous optimisation of the model (and as a result of its outcomes as well) based on the utilization of feedback loop technique that facilitates the nature of ‘self-learning’,
2. Enhanced model outcomes due the usage of surrogate data that enrich the dataset and contribute to better generalization of the proposed model.

In this context, the proposed approach enables the development of personalized the development of personalized value adding services for the SME customers of banks. To this direction, the paper proposes a transaction categorization model that classifies transactions according to the underlying utility of the transaction, and an approach to forecast inflows and outflows based on historical data applied for each general transaction category. The classification of all SME transactions is essential to label all historical data and enable most of the functionality of modern BFM toolkits. Based on the classified data, further personalized analytical tools may be developed which can add value to SMEs by offering a holistic approach to revenue and expense analysis. Regarding the transaction categorization model, a hybrid approach is proposed, by applying a rule-based step approach based on a set of elements including transaction, account and customer data aggregation, following an implementation of a tree-based ML algorithm for the remaining uncategorized transactions. Based on the above, the Transaction Categorization model’s ML algorithm is built on a Catboost model [7] and revolves around a Gradient Boosting Classification task, aiming to produce robust results, with the ability to detect occurring anomalies in several ways. Most popular implementations of gradient boosting use decision trees as base predictors. Even if it is convenient to use decision trees for numerical features, in real case scenarios, most datasets include categorical features, which are also important for predictions. To deal with categorical features, gradient boosting is converting them to numbers before training. Catboost leverages a new gradient boosting algorithm that successfully handles categorical features and benefits from dealing with them during training as opposed to preprocessing time.

To the best of the authors knowledge, there is no open-source model or data available for the specific case of SMEs transactions categorization. Given that lack of prominent research, the utilized dataset , that is provided by a commercial bank and consisted by real-life data was labeled based on various rules, incorporating various internal categorical values present in the dataset. Consequently, the resulting dataset, consisting of 20 ‘Master Categories’ tailor-made for SMES, was highly imbalanced as in most multiclass classification problems where some classes have significantly smaller instances compared to other classes. Many conventional machine learning algorithms suffer from this uneven distribution of data instances among classes and make it less effective in predicting instances of minority class. Although these types of problems belong to an active research area, in our case the Catboost [7] model was preferred based on the findings of [8], where numerous boosting models were evaluated and compared on multi-class imbalanced datasets, showing that CatBoost algorithm is superior to other boosting algorithms on multi-class imbalanced datasets.

The latter task, Cash Flow Prediction refers to an accurate probabilistic time-series model which can predict cashflows of SMEs per giver category. In this case, deep learning techniques were utilized to provide information regarding the future cash flow of SMEs. The proposed framework, as part of a holistic BFM tookit integrates a continuous learning approach that considers the SMEs transactions represented as timeseries able to capture rare market events with very short training time by utilizing probabilistic forecasting based on auto-regressive recurrent neural networks. The techniques mainly focus on time-series probabilistic forecasting, utilizing a Recurrent Neural Network model To this end, a DeepAR model is leveraged within our research. A DeepAR Estimator implements a Recurrent Neural Network (RNN) based model, close to the one described in [9]. More specifically, the DeepAR Estimator introduces a methodology for producing accurate probabilistic forecasts, based on training an autoregressive RNN model on many related time-series. An RNN is a feed-forward neural network that has an internal memory, with the ‘Recurrence’ explaining the fact that the produced output is copied and sent back into the RNN as an additional input. For making a decision regarding every output of every layer, it considers the current input and the output that it has learned from the previous input. Concerning this task, many challenges had to be addressed, with the one with the greatest scientific impact being the usage of synthetically produced surrogate data as a way of enriching the original dataset to improve performance of the underlying deep learning models.

The remainder of the paper is organized as follows: "Related work" section presents related work done in the areas of study of this paper, while "Proposed approach" section delivers an overview of the proposed methodological approach, introduces the overall architecture and offers details regarding the datasets used and how these are utilized within the models. "Evaluation results" section dives deeper into the results of the conducted research and the implemented algorithms, with the performance of the proposed mechanisms being depicted in the results and evaluation section. Moreover, "Evaluation results" section also refers to the explainable AI concept, where LIME and SHAP models are utilized along our classification results. "Conclusion" section concludes with recommendations on future research and potentials of the current study.

## Related work

With regard to transaction categorization in the banking industry, there are plenty of commercial applications based on ML classification approaches, focusing mainly at personal transaction categorization solutions. A related research comes from [10], where short texts were utilized towards a novel system that combines Natural Language Processing techniques with Machine Learning algorithms to classify banking transaction descriptions for usage in a Personal Finance Management (PFM) application. A labelled dataset with real customer transactions was utilized exploiting existing solutions in spam detection by proposing a short text similarity detector to reduce training set size. While in [11] a novel approach is proposed which is utilizing customer transaction data aiming to monitor customer behavior prediction without any manual labeling of the data. To achieve this goal, elements of the banking transaction data are automatically represented in a high dimensional embedding space as continuous vectors. In this new space, the distances between the vector positions are smaller for the elements with similar financial meaning. Likewise, the distances between the unrelated elements are larger, which proves useful in automatically capturing the relationships between the financial transaction elements without any manual intervention. In the same direction, another research [12] presents a novel application of representation learning to bipartite graphs of credit card transactions in order to learn embeddings of account and merchant entities.

Recently, transaction Categorization research revolves around text identification and word embeddings. Recent approaches try to extract latent information from the unstructured text of the description. One representative research is [13] where a framework, is proposed to understand changing trends and take appropriate decisions based on the description of the transactions. Another relevant proposition comes from [14], where a machine-learning-based system automates the process of mapping financial transfers to the corresponding accounts.

Furthermore, various more researches based on more traditional supervised models can be found in the pertinent literature such as [15, 16]. The former approach investigates supervised learning based methods to infer the identity of the sending Financial Institute from the description string of a money transfer transaction, using a blend of traditional and RNN based NLP methods. While the latter presents a research conducted at Norwegian University of Science and Technology trying to answer 3 research questions, including comparison of different ML models, evaluation of external semantic resources like the Norwegian database Brønnøysundregisteret and Google Places API, or linked open data sources like DBPedia and WikiData. The main results of the approaches denote that the semantic resources improve the accuracy of the classification system, while Wikidata and DBpedia lead to a decrease in accuracy [16]. Apart from the research referenced here, there are also various Google patents (i.e. “A transaction management system” and “Categorization of non-contiguous transactions”) including a database system configured to receive and store data for a plurality of financial transactions and a method for improved management of transaction data from a financial services computer network. On the other hand, to the best of the authors knowledge, there is no transaction categorization research for SMEs in the pertinent bibliography. The aforementioned papers justify the intuition behind our transaction categorization approach.

Regarding the time-series forecasting task, there is another related approach of applying probabilistic forecasting referring to potential food recalls [17]. Of course, the time series prediction is one of the hottest sectors in the research literature. In [18], Facebook’s Prophet is introduced, a modular regression prediction model with interpretable parameters that can be adjusted intuitively by analysts familiar with the time series field. In [19] it is compared with DeepAR model in order to forecast food demand, illustrating promising results. However, the results were less than satisfactory in predicting cashflows in our scenario. It is argued that the improved performance of DeepAR in our research comes from groupings per category as implemented in the hybrid classification task, where a global model taking into account all categorization groups before implementing time-series forecasting outperforms other models presented in the pertinent literature. Towards that direction, a systematic review of 117 time-series related papers, comes to strengthen this claim [20]. Additionally, it’s worth mentioning that in the same review, a comparison between some of the most prevalent approaches for time-series forecasting showed that SARIMA is the only statistical method able to outperform (in some cases) the following machine learning algorithms: ANN, SVM, and kNN-TSPI, but without statistically significant difference [20]. Subsequently, no statistical method was leveraged in our research, instead, the GluonTS framework [21] was utilized as a probabilistic Deep Learning framework for time series forecasting. However, the increased forecasting accuracy comes at the expense of a larger number of parameters [20]. Taking into account the latter, the high scale requirements and the widely different magnitudes of SMEs time-series, SARIMA model was not opted. Instead, we used the GluonTS framework as a deep learning library that bundles components, models and tools for time series applications such as forecasting. Moreover, it should be noted that for time series forecasting, the task of using observed time series in the past to predict values in a look-ahead horizon gets proportionally harder as this horizon widens [4].

Numerous studies regarding the effectiveness of AI techniques in the financial and specifically the banking sector denote that the automation and digitization of banking services presents large economic advantages. For example, in [22], the authors examined the influence of AI on the market and start-up companies. An exciting outcome of this paper is that only 6.6% of countries apply AI in their business. In [23], there is a comparison between two algorithms of artificial intelligence; neural network and fuzzy logic. The algorithms predicted the stock market proving that the neural network gave more accurate predictions than fuzzy logic. In [24], the authors applied AI to banking data to check its efficiency on data fraud and customer retention. Another task that AI can help with is customer profiling. For example, in [25], using both supervised and unsupervised learning predicted customer profiling behavior in a Taiwan bank. Furthermore, in [26], there is an extended research regarding the advancements of AI in the banking industry. In Jordan, the [27] study distributed (195) questionnaires to 13 commercial banks. The main results can be summarized in that the neural networks used by Jordanian commercial banks contribute to increasing the efficiency of its accounting systems, and providing management with basic accounting information. The authors discussed [28] developing artificial intelligence in banking services and surveyed to discuss the opinion of the customers. Most opinions supported applying AI in the bank. The study suggested applying the drive-thru bank, automatic Passbook printing kiosks, and chatbots.

The main difference of the proposed work compared to the aforementioned ones is that both the transaction categorization model, as well as the cash flow prediction one, revolve around the specific needs of SMEs and their specific business needs. Moreover, the two developed approaches are interconnected with changes in the categorization process being reflected in the cash flow prediction model. Specifically, this paper addresses the emerging challenge of automated Business Financial Management for SMEs by introducing a data-driven approach to facilitate timely decision making. In this context, the operational objective/contribution is twofold:

1. Timely transaction categorization based on efficient classification of each transaction according to the underlying expense category. Providing a sound approach for how structured unlabelled tabular data can be classified using a Hybrid Model and represented in the time domain as time-series enabling the exploit of the forecasting method.
2. Systematically forecasting expenses based on historical data applied for each general transaction category. To the end, the utility of surrogate data as a dataset enrichment approach was texted and exploited.

The main contribution of this paper is a complete framework leveraging a ML and DL approaches for SMEs financial management. The framework consists of two major components, a classification mechanism relying on a Tree based boosting technique, classifying the transactions according to the implicated product/merchant and a time-series forecasting method consuming the time-series defined from the classified transactions, predicting future cashflows. The main difference of the proposed work compared to the existing ones, is the introduction of a self learning hybrid (i.e. rule-based and ML) approach to map the problem as a time-series case. More specifically, the approach includes: (i) the development of a hybrid transaction categorization model, that offers continuous optimisation and adaptability, by the feedback loop. (ii) Given that for ‘small’ sized time-series datasets, finding an ML/DL forecasting model that offers qualitative forecasts is a major challenge in machine learning as stated in [11], the presented approach proposes the utilization of surrogate data as an enhanced approach providing more accurate results.

## Proposed approach

The main research challenges addressed in this paper enable the classification of SME transactions and the generation of accurate cash flow predictions per category. These two research topics are interconnected as the forecasting model is based on the results of the prior categorization/classification. However, these tasks pose different challenges and methodological approaches, both in terms of data aggregation and preprocessing, as in terms of utilizing and extending prevalent ML/DL techniques. In order to address the various challenges and research achievements, it is key to present the data used for this research, which were obtained by the Bank of Cyprus in order to realize a real-world case scenario.

### Datasets

To the best of the authors knowledge, there is no open-source dataset including SMEs transactions. In the frame of our research, the utilized dataset had been provided by Bank of Cyprus as a real-world use case, composed by tokenized transaction attributes of the bank’s clients (i.e. SMEs). The provided data was represented in a tabular format and were available within the INFINITECH H2020 European Project (GA 856632). These data include transactions, customers and accounts data of over a thousand SMEs for the years 2017 to 2020, exceeding 3,5 million data entries and 3GB in terms of storage. As many variables were included in the datasets obtained by the bank, the main categories and variables/features utilized in our approach are presented in the Table 1 while the categories of the classified transactions that they were depicted in Table 2.

**Table 1:** Provided data sources by Bank of Cyprus

Datasets	Utilized variables/features
Transaction data	Account Holder Key Identifier, Transaction Code, Transaction Date, Original Amount, Currency Code, Amount, Transaction Description, Transfer Account Key, Channel Code’, Merchant Name, Mechant Code’, Debit/Credit Indicator, Merchant Category Code ID
Customer data	Card Holder Key Identifier, Customer NACE code (rev2), Number of Employees, District
SME Accounts Data	Account and Card Keys identifiers
NACE Rev. 2	Open-source NACE codes

**Table 2:** List of master categories (i.e. outputs of transaction categorization)

Uncategorized expense	Utilities	Professional fees	Cash
Selling and distribution expenses	Offices Expenses	Banking Expense	Saving
Taxes	Rent & Lease	Repairs & Maintenance	Transfer
Insurance	Motor Vehicles Running Cost	Sundry expenses	Supplier Expense
Payroll & Benefits in Kind	Subscriptions Donations Expenses	Income	Assets

All SME data were anonymized prior to acquisition, except from the various internal codes kept by the bank. Although there are many internal tokens, not all transactional data is categorized without an initial reference to its base class.

The primary data source for both the transaction classification and cash flow forecasting models was the available transaction data, including over 40 variables/characteristics represented by key information such as dates, amounts, credit indicators/debits, descriptions, etc.. More sophisticated information such as contactless payment indicators, merchant category codes and standby order indicators

The SME Accounts dataset contains symbolic information about a customer’s individual accounts, available balances and corresponding NACE Rev2 codes, which are statistical classifications of economic activity used in the European Community [29] also used by the bank for the identification of the SMEs operating sector. The availability of SME economic activity is also important in this and in our future work, external national and European data that horizontally use the NACE code system can be leveraged to provide more individual sectoral information in our model.

Customer data contains the various accounts belonging to each individual SME, as well as the type of the respective account.

The metadata of each transaction includes the timestamp of each transaction, in this approach another dataset of 9494 time series was created, representing the daily number of Amount spent for each account per category for our given time period. Apart from these time series representing the daily expenses, we also generate some covariates (for example Day of the Week, Month of the year, etc.) for individual time series.

### Surrogate data

The use of surrogate data is introduced in this paper to increase the added-value and impact of the utilized deep learning models, since they benefit from the increased diversity and volume of the training data. It is expected that the usage of analogous data will help the generalization of some, if not all deep learning models in the case of time series forecasting.

The most commonly used techniques for generating surrogate data for statistical analysis of nonlinear processes include random shuffling of the original time series, Fourier-transformed surrogates, Amplitude Adjusted Fourier-Transformed (AAFT surrogates), and Iterated AAFT surrogates (IAAFT) [30]. In our work, we incorporated the IAAFT method to address the issue of power spectrum whitening, as the main drawback of AAFT method, by performing a series of iterations in which the power spectrum of an AAFT surrogate is adjusted back to that of the original data before the distribution is rescaled back to that of the original data.As noted by [31], only a few studies investigate data augmentation from frequency domain perspective for time series. The generated time series from AAFT and IAAFT can approximately preserve the temporal correlation, power spectra, and the amplitude distribution of the original time series. That makes these approaches a appropriate candidate for augmentation of time-series. In the experiments of [32], the authors conducted two types of data augmentation by extending the data by 10 then 100 times through AAFT and IAAFT methods, and demonstrated promising classification accuracy improvements compared to the original time series without data augmentation. Furthermore, the same approach was tested on [17], where the usage of IAAFT method yields promising results as timeseries augmentation technique.

Through this proposed approach, with each iteration the change to the distribution that occurs when the Fourier amplitudes are adjusted will be smaller than in the previous iteration, and thus the alteration of the power spectrum when the rescaling is performed will also be smaller than in the previous iteration. In fact, Schreiber [33] showed that, for a nonlinearly transformed autoregression process, the iteration procedure will converge towards the power spectrum of the original data until a saturation point is reached, where the Fourier amplitude adjustment is so small that the rescaling puts the data into the exact order it had before the amplitude adjustment.

### Data-driven models

A decision tree Machine Learning algorithm, able to represent how different input variables lead to predicted target categories, and a probabilistic forecasting Deep Learning model, offering probabilistic forecasts for future inflows and outflows of SMEs per category have been chosen as the most suitable ones based on the pertinent literature, since the problem to be addressed is consisted of a combination of classification (i.e. transaction categorization) and time-series prediction (i.e. cash-flow prediction) tasks. In more detail, the transaction categorization task can be translated as a task of classifying multi class highly imbalanced tabular data, where the Catboost classifier utilized seems to thrive compared to other approaches, as argued in [8]. At the same time, given that the examined time-series case is a multi-step ahead prediction task, it should be mentioned that for time-series forecasting, the task of using observed time-series in the past to predict values in a look-ahead horizon gets proportionally harder as this horizon widens [4]. To this end, a probabilistic approach is preferred, providing a predicted probability distribution accompanied by different confidence levels. The DeerAR is a state-of-the-art model of probabilistic forecasting leveraging a Deep Recurrent Neural Network (i.e. LSTM) for predicting the mean values and the standard deviation, while a Monte Carlo simulation providing the probability distribution of the forecast.

In more detail, the DeepAR Estimator implements an RNN-based model, close to the one described in [9]. More specifically, it applies a methodology for producing accurate probabilistic forecasts, based on training an autoregressive Recurrent Neural Network model on many related time series. RNN is a feed-forward neural network that resembles an internal memory [34]. For deciding regarding every output of every layer, it considers the current input and the output that it has learned from the previous input. Furthermore, our solution proposes a continuous learning approach instead of a classical machine learning pipeline where the model is trained once and for a long time period in a large dataset. Contrastingly, in DeepVaR the model is retrained online on the latest transaction data, addressing the dynamic nature of market-based transaction behavior data while avoiding at the same time model bias and drift.

**Fig. 1:**
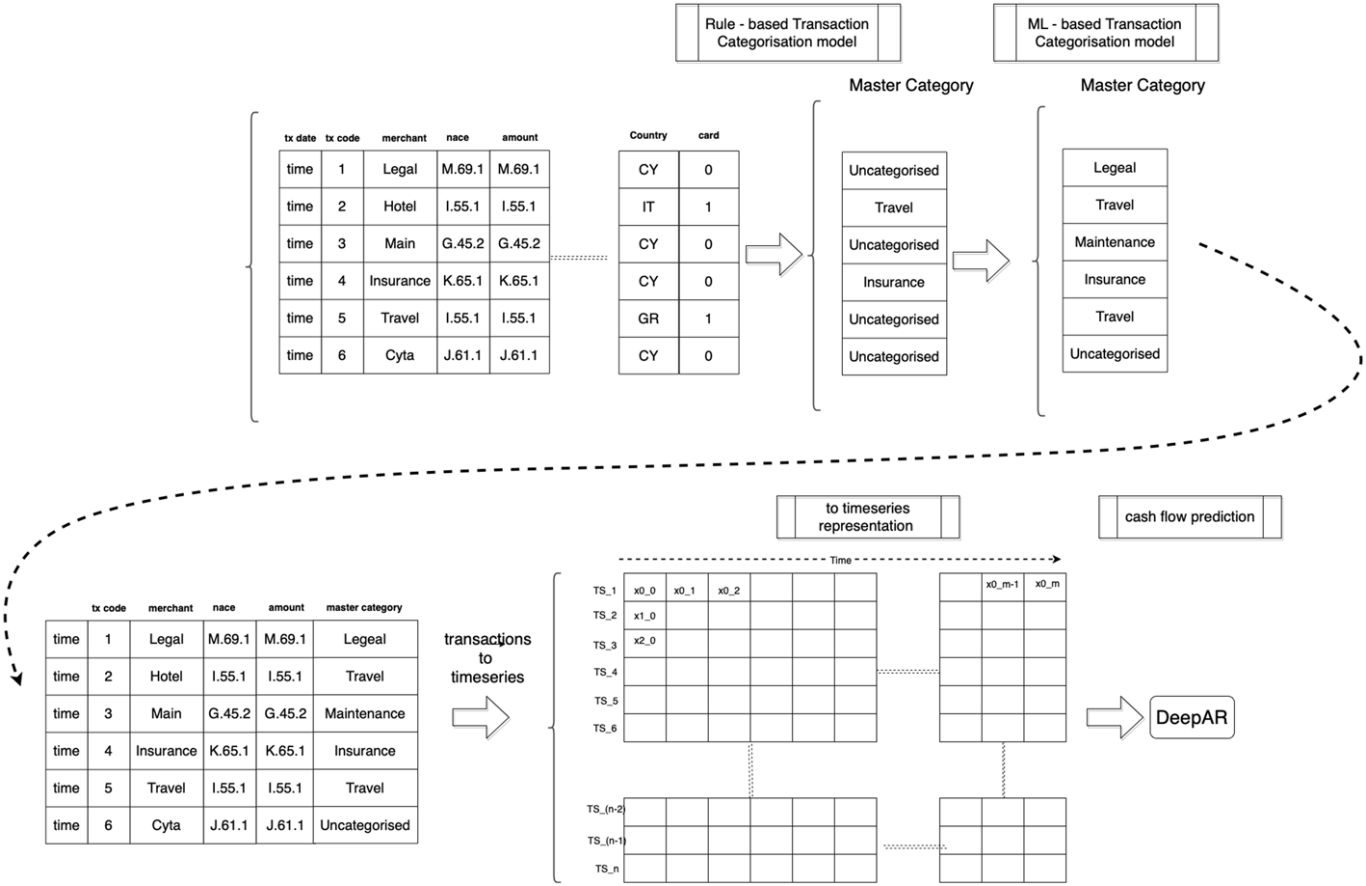
Schematic explanation of the data aspect to the end-to-end approach including the four main steps to the process

A more detailed view of the proposed approach provided in Fig. 1, which depicts the data handling pipeline, in a stepped approach, to apply both the transaction categorization process and the cashflow prediction model. In the first step, the raw data were pre-processed and fed into the Rule based model categorization model. Then the ‘uncategorized’ (i.e. not labeled by Rule base model) transactions were classified in specific categories and finally the data was transformed in time-series representations to be utilised for prediction of future inflows and outflows.

**Fig. 2:**
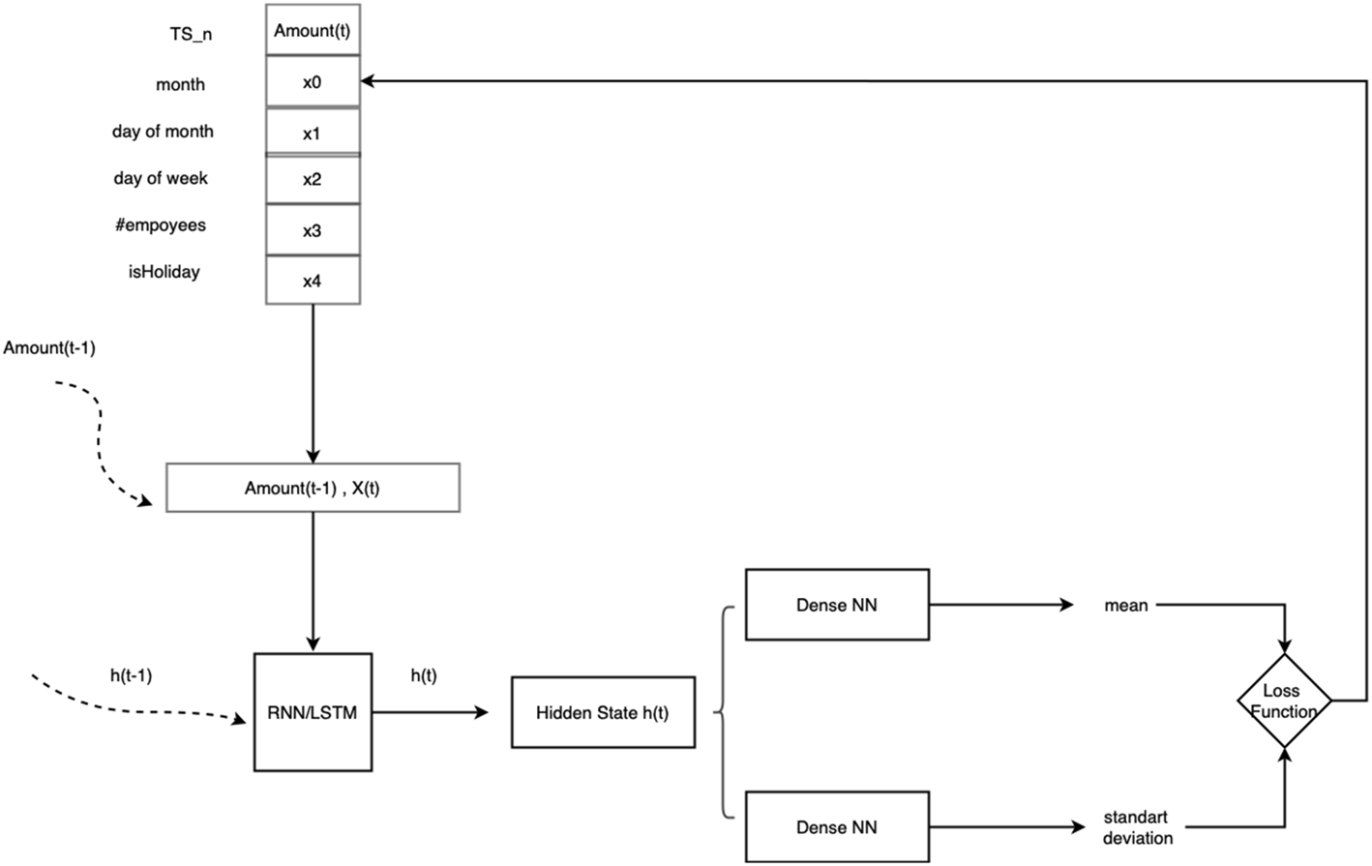
Flowchart of cashflow prediction of inner processes

What is more, the DeepAR model learns seasonal behavior patterns from given covariates which strengthens its forecasting capabilities ,with inner processed illustrated in Fig 2.

In addition, we have included Algorithm 1 to better illustrate the transaction categorization process.

The stationarity of the data was checked by using augmented Dickey-Fuller test [35]. This process is of major importance for any predictive method that exploits historical data since these methods are usually based on the assumption that the data generation mechanism does not change over time. Furthermore, it should be noted that in terms of predictions, the prediction time-frames of the recalls may be over 1/4 of a year (i.e. 3 months). These predictions aim at improving the internal financial monitoring of an enterprise and its various inflows and outflows, while also enabling professionals to ensure the business continuity of their clients (i.e. SMEs), minimize future risks and financial losses. Based on the latter, three options stand out:

1. Use the dataset as is, with its daily frequency resulting in a 90-time-step window of prediction. This is not recommended as most of the time-series produced are sparse leaving no obvious pattern to be learnt from.
2. Resample the data in weeks, resulting in a 16-timestep window of prediction. While some patterns begin to emerge, the data in some cases are still very sparse and the cumulative error from the 16-step prediction is theoretically relatively big.
3. Resample the data in months periods, providing stationary time-series with visible patterns and a lower theoretical accumulated error of prediction, since the window has been reduced to 3-timesteps.

**Fig. 3:**
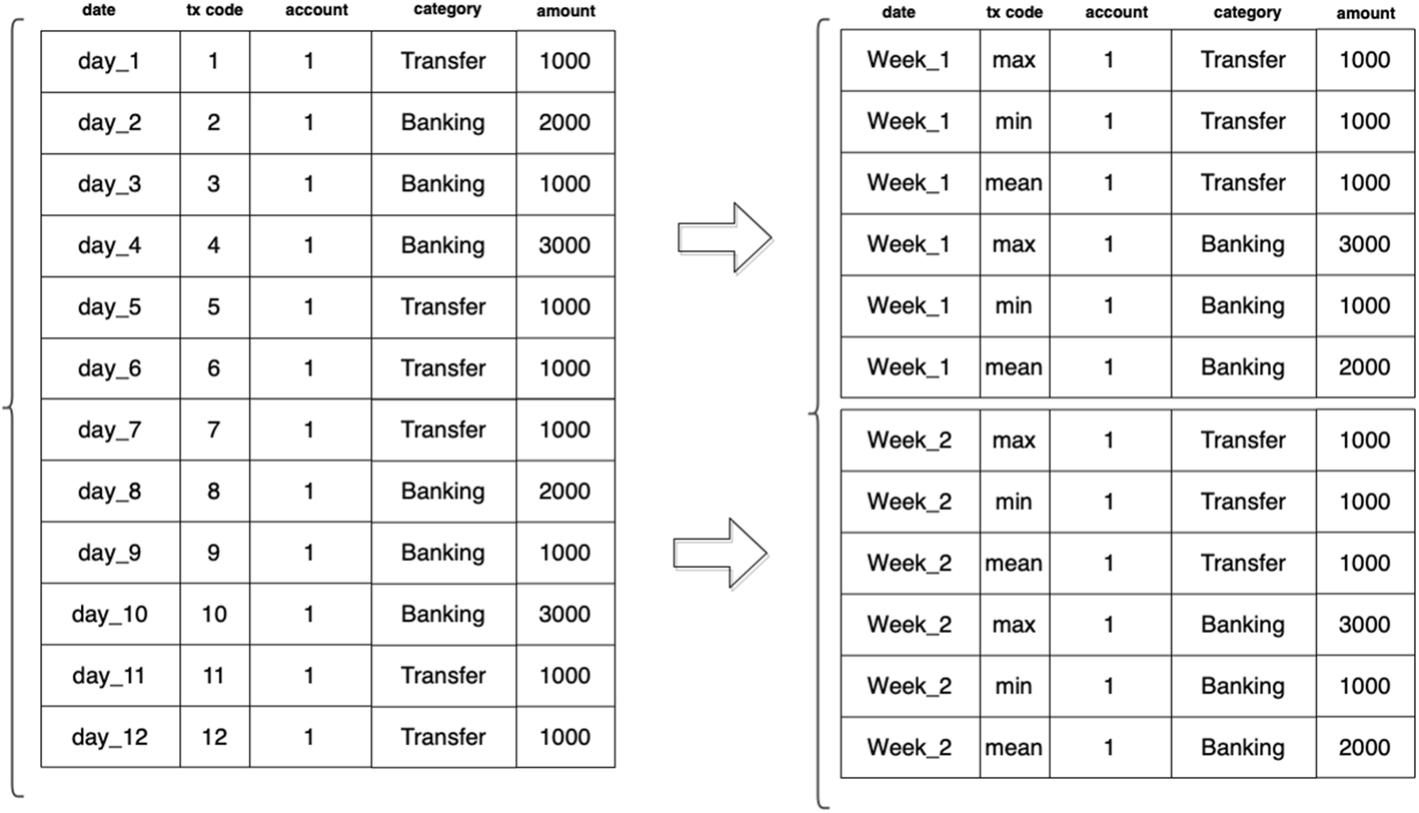
Example of resampling from days to weeks timeframe

The data were re-sampled in weekly time frame by calculating the aggregate amount of inflows and outflows respectively per account as depicted in Fig 3. The time frame of “months” was selected as it corresponds to the data velocity for the given case—i.e. the strategy of the SMEs can be projected in a 4-month horizon.

The Catboost Classifier [7] is a gradient boosting ML model based on decision trees, which has the ability to handle the categorical features by nature. CatBoost framework has implementations either based on CPU or GPU which optimizes its performance in terms of time CatBoost as gradient boosting algorithm consists of two phases when building trees The first phase results to the selection of the tree structure and the second one determines the number of leaves for the tree. One significant improvement of the CatBoost is that it enables the unbiased gradient estimation in order not to overfit. To this end, in every iteration of the boosting, for calculating the gradient of a sample, it excludes that sample from the training set of the specific decision tree model. Another improvement refers to the automatic encoding of categorical features to numerical features without any preprocessing phase.

**Fig. 4:**
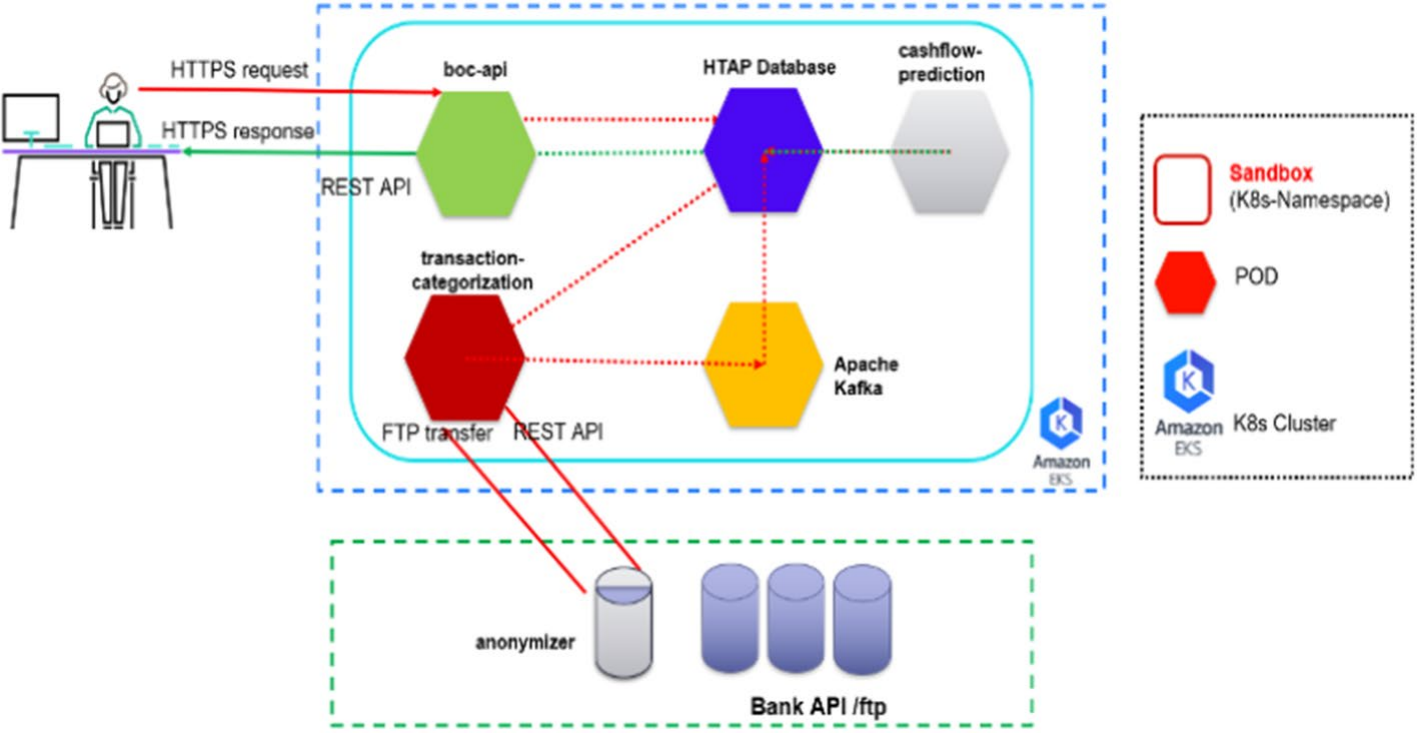
Conceptual architecture of the proposed approach

Our goal is to utilize the above mentioned ML/DL models to enable explanatory insights by leveraging available SMEs financial transactions in order to track and optimize their financial behavior. The conceptual architecture of the proposed mechanism is depicted in Fig. 4

## Evaluation results

**Fig. 5:**
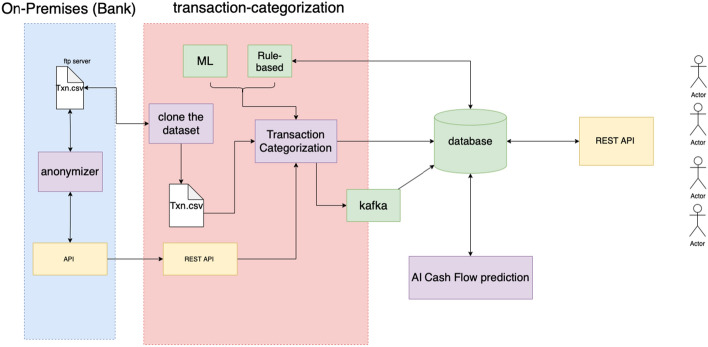
Logical view of the connected components of the framework including transaction categorization and cashflow prediction

A description of the evaluation strategy regarding the approaches introduced in this paper is initially provided, followed by the results of both the transaction categorization and the cash flow prediction models. A more detailed view of this is provided in Fig. 5 that depicts the logical view of the connected components along with the Fig. 3, which depicts the data handling pipeline, in a stepped approach, to apply both the transaction categorization process and the cashflow prediction model. In the first step, the raw data were pre-processed and fed into the Rule based model categorization model. Then the “uncategorized” (i.e. not labeled by Rule base model) transactions were classified in specific categories and finally the data was transformed in time-series representations to be utilised for prediction of future inflows and outflows.

### Hybrid transaction categorization model

As mentioned above, the absence of labelled data is the main challenge when developing a transaction categorization model. In these scenarios, two prevalent approaches arise. The first one is based on unsupervised ML models to create clusters with no prior knowledge of the expected outcomes. However, as labels are fixed and non-negotiable in the finance sector, this approach may lead to a less robust model in terms of confidence level, with some clusters not being able to reflect a distinct category with real-world value to the SME. The second one (here lies our approach as well) is to first label a representative subset based on expert knowledge, creating a rule-based model, which can then be integrated with a supervised machine learning model, with a high degree of updated automation enabling the transaction reclassification. To this end each part of the Hybrid Model (rule-based model, ML model) has to address specific challenges. Specifically A step approach was followed for the rule-based model, incorporating various internal codes of the Bank, some of them being interpreted only by the specific banking employees (i.e. Transaction Type Code) and others being used universally in the business world (i.e. Merchant Category Code and NACE codes) [36]. In more detail, the main variables used are illustrated in the Table 3.

**Table 3:** Dataset description

Variable	Description
Transaction Type Code	An internal code provided for every bank transaction, providing information regarding the transaction type and including Root Categories like cash withdrawals, deposits, banking fees etc.
Merchant Category Code	A four-digit number listed in ISO 18245 for financial services, mainly used to classify a business by the types of goods and services provided
NACE code	A European industry standard system used across European Union for classifying business activities. Utilizing the NACE code of the transfer account found in the available SME customer data
Transaction description	Including mainly native retailers, service providers, consulting and legal firms not included in previous steps

**Fig. 6:**
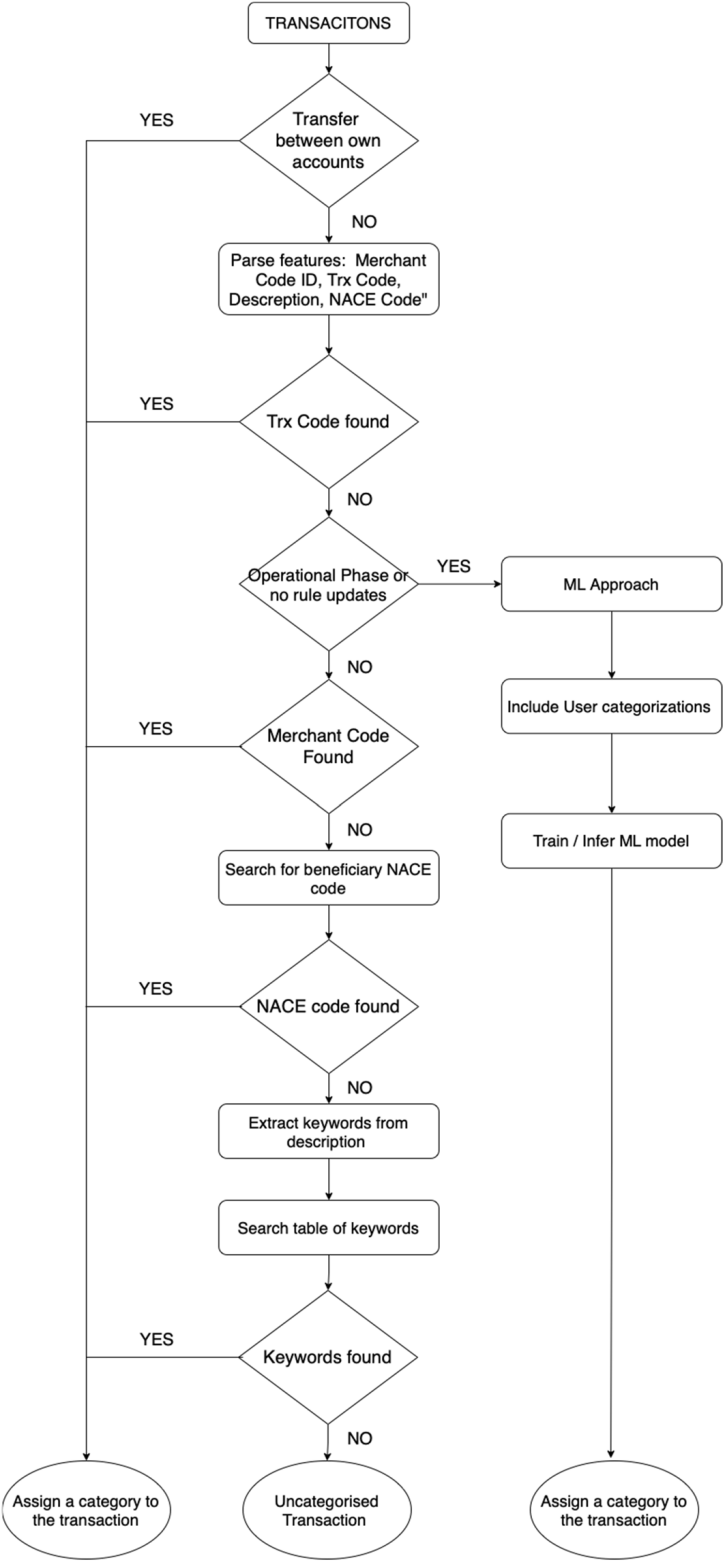
Hybrid classification model flowchart

**Fig. 7:**
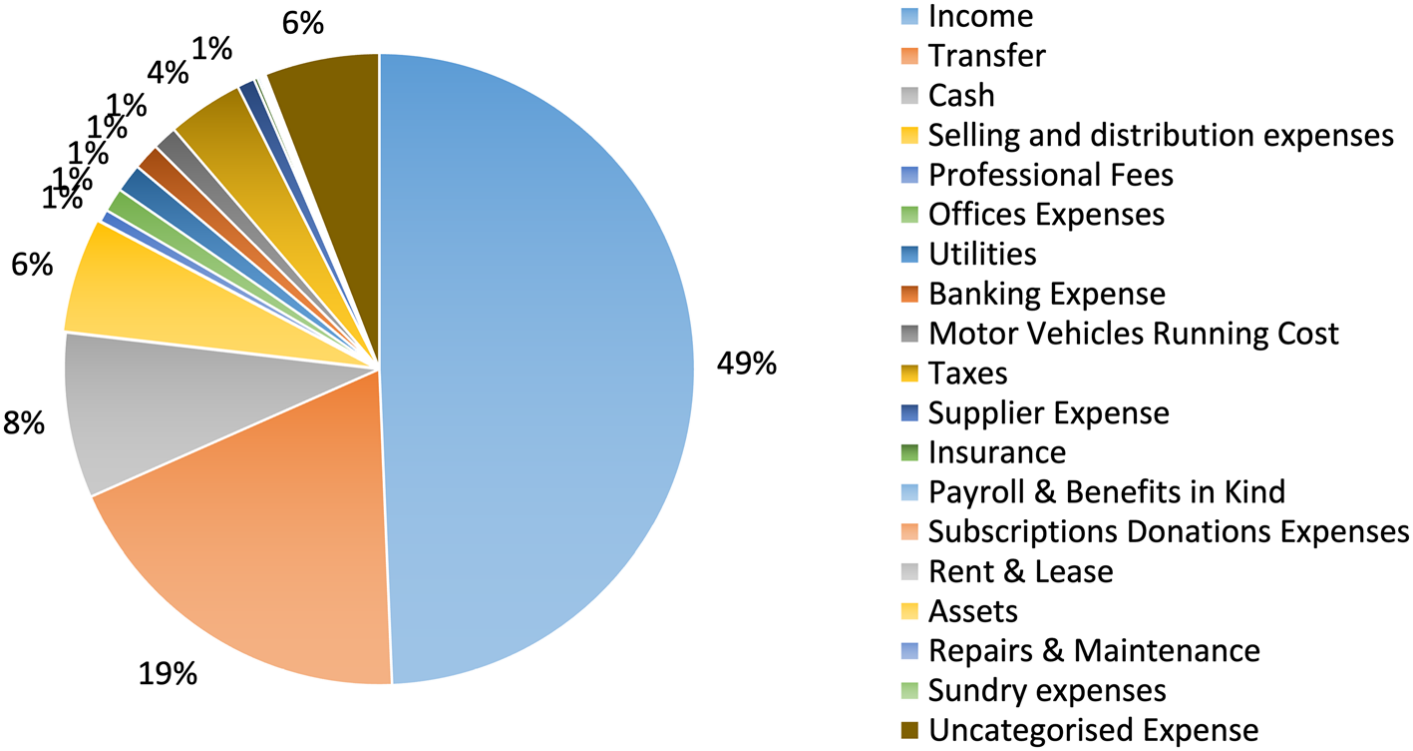
Rule based step approach categorization results

Before assigning the variables above to given categories, it was important to capture all transactions between the same accounts of the same SME, since those would be classified as ’Transfers between own accounts’. The exact flowchart of the hybrid transaction is illustrated in Fig. 6, depicting both the rule-based step categorization and the ML classification approach. The categorization evaluation is depicted in Fig. 7 which is referring to the distribution and the coverage of the dataset that can be accomplished based on the rule-based step approach.

As far as the Catboost Classification model is concerned, a key challenge in developing an effective hybrid classification model is to reduce the bias by the rule-based model, allowing the user to re-categorize a given transaction category. This process of updating the labels is out of the scope of this paper, however, the way of updating our knowledge and adapting the model to re-categorizations is fundamental. To this end, a Catboost model was trained in a way of adopting to changes made by the SMEs. Given the different steps utilized of the rule-based model and towards high efficiency and categorization accuracy, a hybrid-model was preferred instead of a fully-AI one, as a result of the confidence provided by the categorization based on the first two steps of the rule-based model.

In further detail, because the first two phases of the rule-based model (transfer across accounts and Transaction Type Code mapping) yield fixed root categories, the goal of this model is to learn and duplicate the final three steps. The number of the remaining labels (i.e. Master Categories) that can be produced in these steps are 16, so the evaluation on multi-class tasks in terms of various metrics was applied. In order to have a better notion of the result and the predictive power in each label, a confusion matrix is presented in Table 4. The table refers to the results on the test set considering a 40-30-30 evaluation schema, where 40% is the training set 30% the validation and 30% the test set. The results presented have been obtained from a Catboost model that has been already optimized in terms of hyper-parameters, fine-tuned using grid-search techniques and intuition, as a fundamental step of a ML pipeline. Given the labels’ significant imbalance, finding a suitable normalizing factor was another problem addressed via hyper-parameter optimization. Grid-search and Random-search algorithms are widely used for hyperparameter tuning. Basically, the domain of the hyperparameters into a discrete grid. Then, every combination of values of this grid (i.e. grid-search) is used in a cross-validation evaluation schema. The point of the grid that minimized the average loss of the cross-validation, is the set of values for the hyperparameters that are used in our model. The process for the transaction categorization is summarized in Algorithm 1.

**Table 4:** Confusion matrix of transaction categorization multiclass classification

		True values															
		**0**	**1**	**2**	**3**	**4**	**5**	**6**	**7**	**8**	**9**	**10**	**11**	**12**	**13**	**14**	**15**
Predicted values	Assets	566	0	0	0	0	0	0	0	0	0	0	0	0	0	0	0
	Banking Expense	0	245	0	0	0	0	0	0	0	0	0	0	0	0	0	0
	Insurance	0	0	1325	0	0	0	1	0	0	0	0	0	0	0	0	0
	Motor Vehicles Running Cost	0	0	0	1256	0	0	0	0	0	0	0	0	0	0	0	0
	Offices	0	0	0	0	15321	0	59	0	0	7	0	0	0	57	0	0
	Payroll Benefits in KInd	0	0	0	0	0	958	1	0	0	0	0	0	0	0	0	0
	Professional Fees	0	0	0	0	0	0	31121	0	0	4	0	0	0	260	0	0
	Rent Lease	0	0	0	0	0	0	0	680	0	0	0	0	0	12	0	0
	Repairs Maintenance	0	0	0	0	0	0	0	0	53	53	0	0	0	0	0	0
	Selling and distribution	0	0	0	0	2	0	1	1	0	39751	0	0	0	1061	0	1
	Subscriptions Donations	0	0	0	0	0	0	0	0	0	0	734	0	0	23	0	0
	Supplier	0	0	0	0	0	0	0	0	0	0	0	1365	0	2	0	0
	Taxes	0	0	0	0	0	0	8	0	0	0	0	5	0	1	0	0
	Transfer	0	0	0	0	0	92	0	0	1	5	0	0	0	41177	0	34
	Utilities	0	0	0	0	0	0	0	0	0	0	0	0	0	5	0	0
	Uncategorized	0	0	0	0	0	0	0	0	0	0	0	0	0	0	0	10124


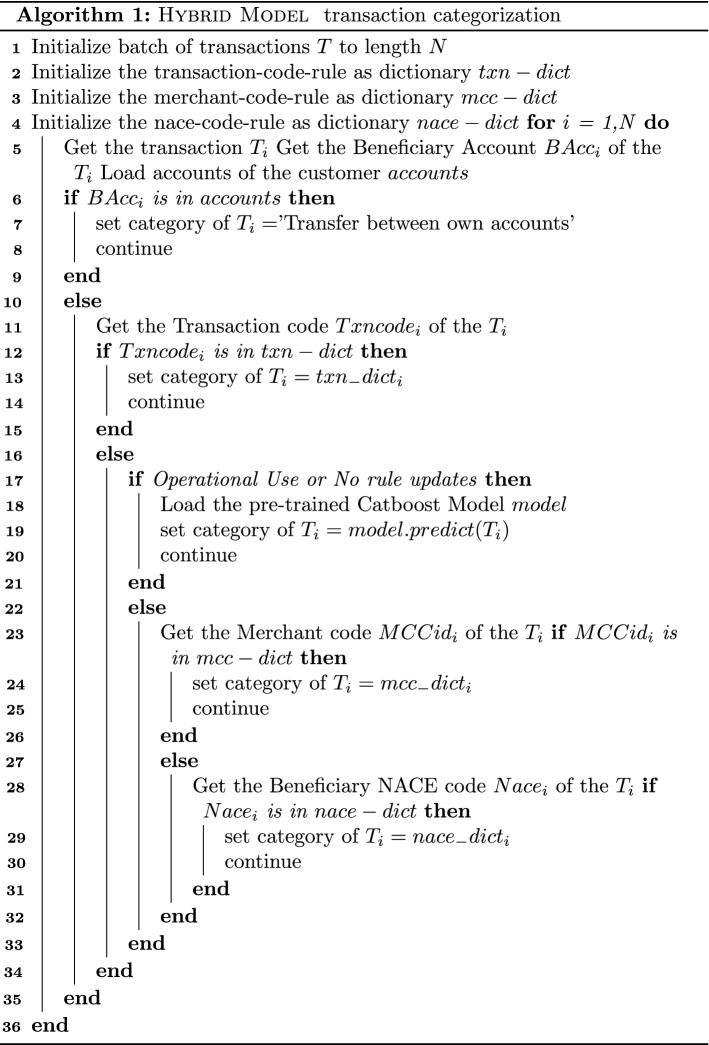


The main outcome is that the model learns the rules which are based on the NACE code and MerchanCode and correctly categorize the results with 98% accuracy. It is worth mentioning that an “Uncategorized Expense” category is included as the transactions falling to this category shall be then categorized by the SME and incorporated to the model.

Although statistics is well established and researched, with its varied hypothesis testing and systematic examination of variable relevance, the same cannot be said for the explainability of various approaches in machine and deep learning. Stronger forms of interpretability could offer several advantages, from trust in model predictions and error analysis to model refinement. This applies better in sectors where heavy-cost decision making is involved such as medicine, or in cases where proactive measures should be taken.

Given the nature of the banking services, the interpretation and comprehension of the results is more significant than the results themselves in order to conclude to both technical and business insights. In general, explainable methods can be categorized in two general classes:

1. Machine learning model built-in feature importance;
2. Post-hoc feature importance utilizing models such as LIME [37] and SHAP [38]. Regarding the transaction categorization, both LIME and SHAP frameworks were leveraged as a qualitative evaluation of the results.

**Fig. 8:**
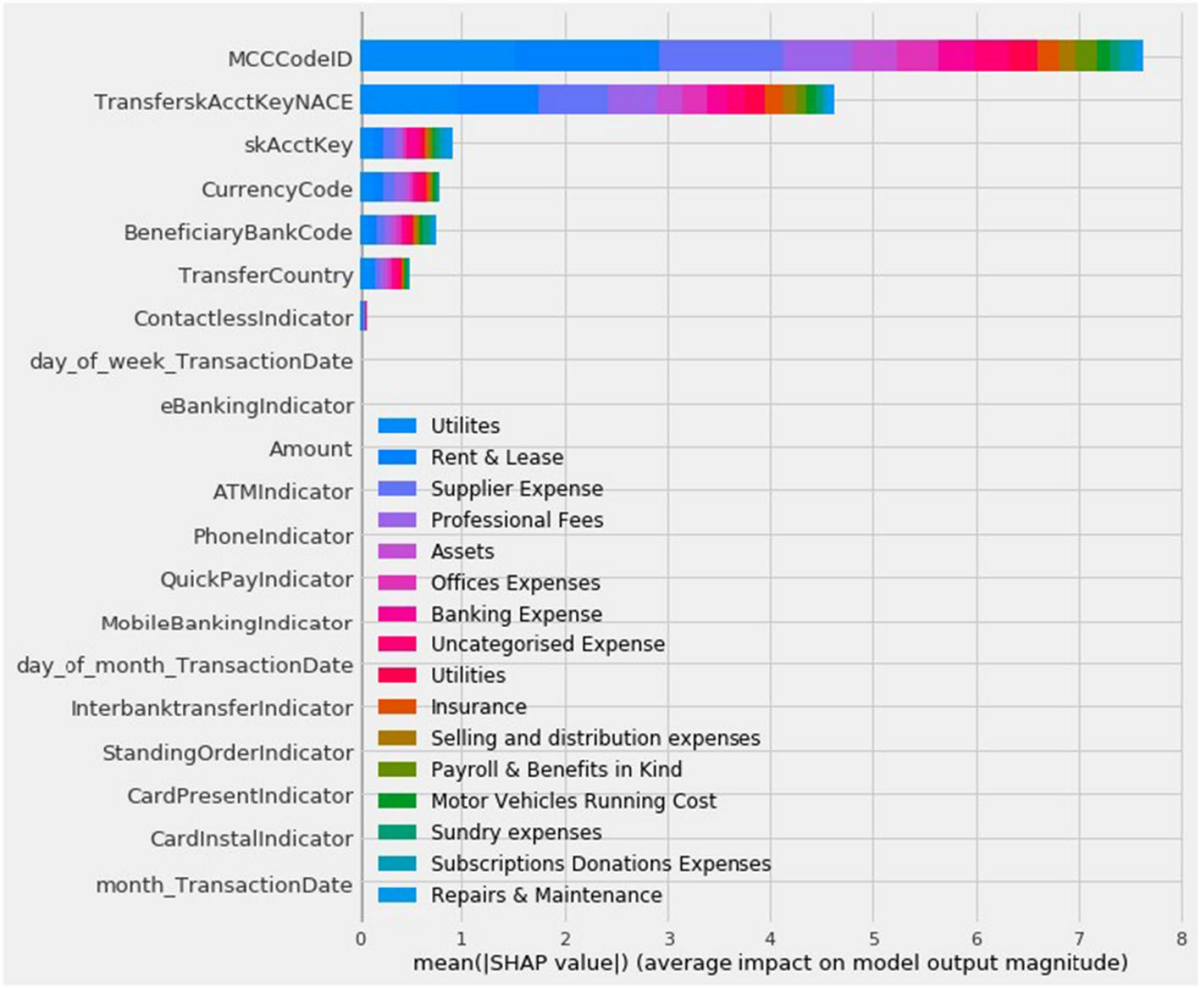
SHAP values indicating feature importance of Cathbost transaction categorization model

Both the LIME and SHAP methodologies were used as a qualitative evaluation of the findings in our transaction classification instance. Fig. 8 depicts the SHAP values indicating the relevance of each feature included in the Catboost model. The image shows that the model learnt the rules that we based on the transaction beneficiary NACE code and the Merchant Code ID (i.e. MCCCodeID), which are the two most significant attributes. Furthermore, the importance of the Account Key (i.e. skAcctKey) as the model’s third most significant characteristic validates the suggested strategy of a user-oriented updating method.

**Fig. 9:**
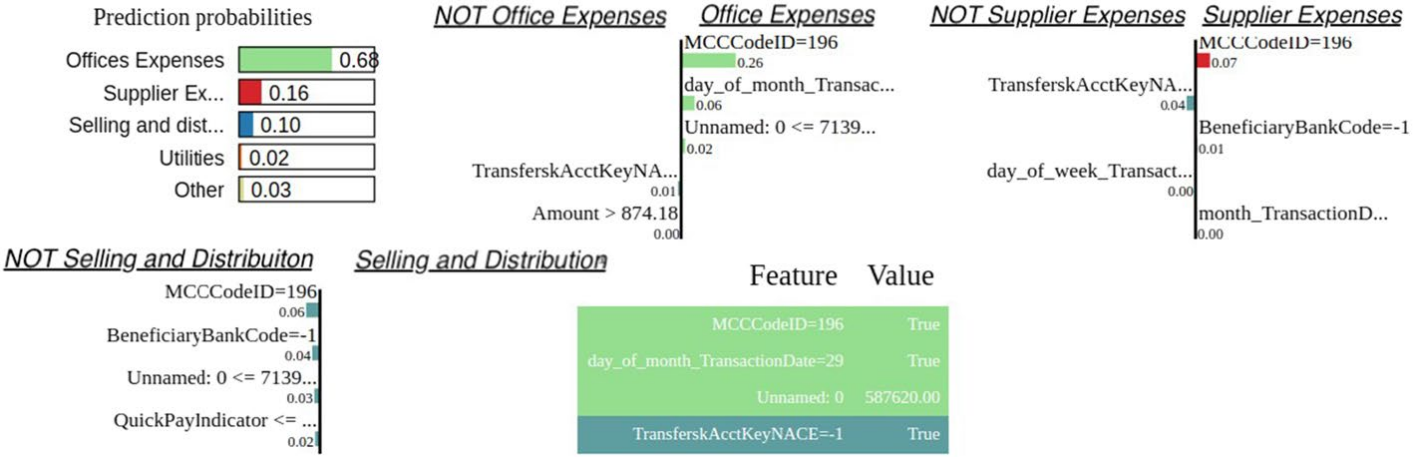
An example of transaction categorization of LIME analysis, explaining the features and their values contribution these specific the outcomes of the model with the provided probabilities

Finally, as far as the Catboost evaluation is concerned, apart from the feature importance based on SHAP analysis, it is of high importance to qualitatively check some of the outcomes based on the Local Interpretable Model-agnostic Explanation (LIME), a recent technique capable of explaining the predictions by using local approximations (sample-based) of models. In Fig. 9 three cases are depicted of how the LIME framework can be of help towards interpreting the predictions of a ML model. For instance, as illustrated in the figure, this transaction is categorized as “Office Expenses” with probability 68% and features contributing towards this outcome are the MCCCode, the day of the month. Likewise with probability 16% it can be categorized as “Supplier Expenses” based on the MCC code the Beneficiary Account and Bank Code, while with probability 10% it can be classified as “Selling and Distribution Expenses”, with the features contributing towards this decisions also being depicted.

### Probabilistic cash-flow prediction model

The aim of this second part of our research is to accurately predict the cashflows per category produced by the hybrid transaction categorization model per SME. The way that the task and the objective were modeled highlighted the need for data transformation in time-series representation. To this end, we resampled and aggregated the amount of the transactions per account and date.

**Fig. 10:**
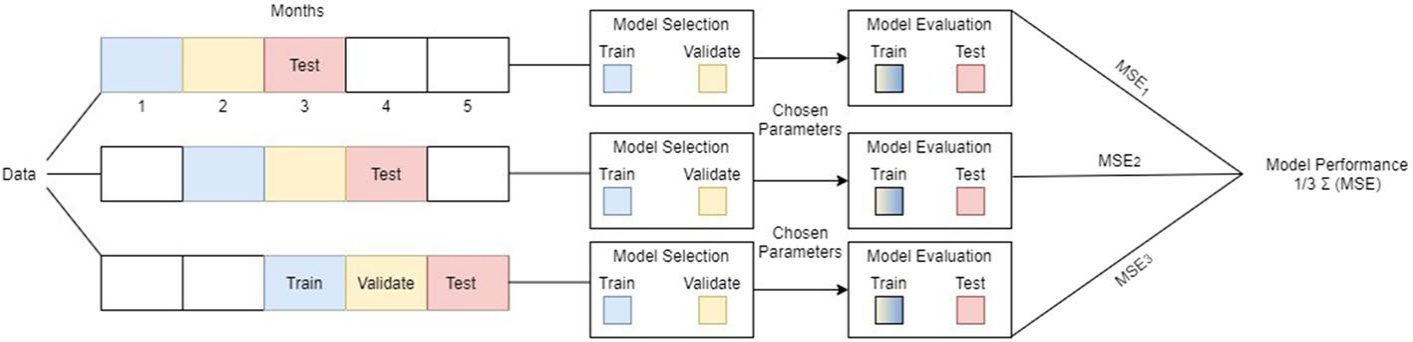
Proposed one step forward validation scheme

Various long-established challenges regarding time-series forecasting arose that should be tackled in the context of our research. These challenges can be summarized as follows:

1. The “cold start” problem, which in this specific case refers to the time-series that have small number of transactions. The ‘cold start’ refers to the difficulty for obtaining accurate predictions for a new customer. As it could be further examined in future research, in order to give a notion, we can argue that in such cases where a new customer account is added in the pool of accounts, the prediction will be based on similar accounts based on the characteristic of the underlying SME (i.e. number of employees, NACE code etc).
2. The stationarity–seasonality trade-off, where it is assumed that in order to have predictable time-series they have to be stationary (i.e. without trend and seasonality factors present). Within the context of this paper, it is argued that this challenge it has been tackled through the leverage of the DeepAR model. As described, the specific implementation of DeepAR utilized addresses these types of problems.
3. The presence of noisy data and/or many observed outliers. The solution to this challenge came from the resample approach used. The scope of the resampling was mainly to reduce the effect of noise in the dataset,while also adding at the proposed model and procedures additional efficiency in terms of time and resources. The time frame of ‘months’ was selected as it corresponds to the data velocity for the given case—i.e. the strategy of the SMEs can be projected in a 4-month horizon.
4. The short length of the dataset hampers the applicability of ML/DL techniques. The final challenge is caused as a side-effect from the fact that the data was re-sampled in weekly time frame getting the aggregation of the Amount of the transactions made for a specific category. This process concludes to the total training data consisting of a matrix $N \in \mathbb {R}^{9494x250}$, where “9494” corresponds to the number of time-series of accounts per transaction category and “250” to the last 250 weeks time-steps of each. In order to produce more reliable results, we trained every model 90 times on a rolling window of 160 time-steps forming 90 tables $\in \mathbb {R}^{9494x250}$ (ex. [9494,1-160], [9494,2-161] etc.). While all models share some hyper-parameters such as $epochs \leftarrow 10$, the $length(prediction) \leftarrow 12$ and $length(context) \leftarrow 24$ months. These specific parameters were fine-tuned using grid-search techniques and intuition, as a fundamental step of the ML pipeline. As far as the surrogate data is concerned, the data used for the creation of the analogous data is the first 160 points, while the number of evaluation steps remains 90, concluding to 90 tables $\in \mathbb {R}^{9494x320}$. To address the transaction length adequacy required to perform predictions, a minimum number of executed transactions during the examined historical period was set as a threshold, with only the time-series illustrating more than ten (10) transactions being utilized.
5. When machine learning is applied, cross-validation methods hide a pitfall in time series forecasting approaches, as they may introduce an overlap between train and test data, known as data leak. Thus, the optimum approach is to simulate models in a “walk-forward” sequence, periodically re-training the model to incorporate specific chunks of data available at that point in time. This procedure is depicted in Fig. 10.

To develop an efficient Transaction Categorization model, various state-of-the-art models were applied and evaluated, with the comparison illustrated at Table 5. It is evident that XGboost outperforms our Catboost model performance, however, as training time is crucial when developing a real-world applicable service, the use of Catboost is considered favorable. Given that the proposed approach was developed in the scope of INFINITECH project as a real-world scenario for Bank of Cyprus and with the purpose to be easily replicated by other banks or researchers, timely model online training consists a key requirement of our transaction categorization model and thus preferred over XGboost as there is a minor F1-score difference. However, as XGboost is considered indeed a viable model selection when offline training is an option, its benefits are included and stressed in our research.

**Table 5:** Comparison of various classification models for transaction categorization task

Model	Accuracy	AUC	Recall	Prec.	F1	Kappa	MCC	TT (Sec)
Extreme Gradient Boosting	0.9554	0.698	0.8012	0.9569	0.9545	0.943	0.9434	298.3
CatBoost Classifier	0.9476	0.6974	0.7398	0.9503	0.9461	0.9329	0.9335	49.64
Extra Trees Classifier	0.9457	0.6949	0.7547	0.9453	0.9447	0.9306	0.9308	15.27
Logistic Regression	0.9427	0.6969	0.7258	0.9458	0.9409	0.9267	0.9275	41.51
Gradient Boosting Classifier	0.9402	0.6944	0.7518	0.9446	0.939	0.9234	0.9243	170.4
SVM - Linear Kernel	0.9396	0	0.6948	0.9446	0.937	0.9226	0.9237	3.268
Ridge Classifier	0.937	0	0.6917	0.9437	0.9342	0.9191	0.9203	0.396
Linear Discriminant Analysis	0.934	0.6953	0.6867	0.9444	0.9333	0.9156	0.9175	8.38
K Neighbors Classifier	0.8455	0.6678	0.4984	0.8341	0.8366	0.8014	0.802	121.1
Naive Bayes	0.818	0.6401	0.7371	0.952	0.8615	0.7776	0.7866	1.359
Ada Boost Classifier	0.7951	0.5463	0.4443	0.6992	0.7353	0.7255	0.7456	3.36
Light Gradient Boosting Machine	0.6293	0.5559	0.3534	0.6996	0.6429	0.5442	0.5484	20.12
Quadratic Discriminant Analysis	0.0111	0.354	0.0496	0.2889	0.0203	0.0068	0.031	4.846

Results 10-fold Cross-validation (with bold noted the proposed model)

Another interesting results seem to be the difference in performance of DQA compared to Tree based models. The worst classification result was obtained with the QDA algorithm with 0.01% accuracy.

Quadratic Discriminant Analysis is a classifier with a quadratic decision boundary, generated by fitting class conditional densities to the data and using Bayes’ rule. Quadratic discriminant analysis (QDA) models the likelihood of each class as a Gaussian distribution, then uses the posterior distributions to estimate the class for a given test point. The method is sensitive to the knowledge of priors. Despite being a popular choice for classification, QDA does not perform very well when the class-conditional probability densities are very different from the normal distribution. This is also noticed on other tasks such as NLP tasks [39].

**Table 6:** Average results per transaction category based on various metrics

Category (expenses)	Seasonal error	MASE	sMAPE	MSIS	Quantile Loss [0.5]	Coverage [0.5]	Quantile Loss [0.8]	Coverage [0.8]	Quantile Loss [0.95]	Coverage [0.95]
Assets	425.004	0.955	1.940	26.064	2401.091	0.466	2900.684	0.722	2367.565	0.871
Banking	694.784	72.957	1.910	2478.095	3340.891	0.477	4437.331	0.746	4268.931	0.891
Cash	4065.432	0.787	1.658	18.493	25428.835	0.515	31496.964	0.755	26010.145	0.882
Insurance	156.242	1.018	1.921	23.230	964.912	0.464	1150.166	0.724	908.232	0.872
Motor Vehicles Running Cost	600.610	3.625	1.702	50.167	3599.007	0.442	3863.190	0.730	2646.931	0.880
Offices	249.595	3.975	1.829	57.882	2379.410	0.470	3007.474	0.736	2660.359	0.879
Payroll & Benefits in Kind	382.469	1.972	1.940	46.086	3326.830	0.468	4666.329	0.730	4582.028	0.874
Professional Fees	2117.501	7.830	1.818	265.033	16769.57	0.480	19810.913	0.743	15255.594	0.884
Rent & Lease	803.825	0.938	1.944	15.177	4093.789	0.465	4920.855	0.709	3708.97	0.856
Repairs & Maintenance	210.978	7.209	1.945	54.962	1815.282	0.487	2171.715	0.740	1693.385	0.879
Selling and distribution	773.484	5.174	1.683	110.461	4951.311	0.4901	5679.797	0.751	4288.930	0.887
Subscriptions Donations	74.533	25.023	1.920	215.479	916.44	0.472	1052.954	0.7314	776.814	0.880
Supplier	2107.319	2.459	1.849	44.455	18531.24	0.463	19777.18	0.726	14281.448	0.872
Taxes	2786.071	0.403	1.825	6.073	13210.548	0.366	12938.64	0.5712	8515.301	0.801
Transfer	8942.757	3.394	1.577	116.278	57575.255	0.494	63056.221	0.751	43652.938	0.888
Uncategorised	11734.934	2.673	1.634	59.273	72403.651	0.483	76042.949	0.741	49743.826	0.881
Utilities	291.783	4.429	1.848	76.449	1855.911	0.468	2126.565	0.731	1604.632	0.878

**Fig. 11:**
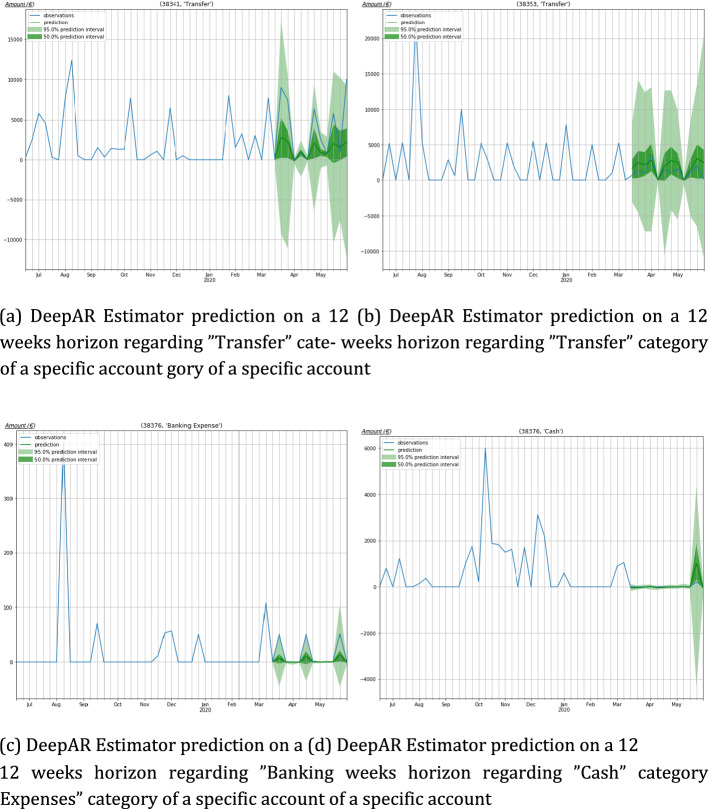
Indicative examples of estimator on specific time series, in each time diagram the blue line represents the real values while the green gradient depicts the predictions in 50% and 95% confidence levels

In Table 6 the performance of the cash flow prediction model is presented in terms of various evaluation metrics. In detail, seasonal error, MASE, sMAPE, MSIS and Quantile Loss are depicted, with the Coverage provided by different confidence levels being presented as well. The results refer to average values for all the incorporated accounts per transaction category. Furthermore, the relevant plots are depicted in Fig. 11, illustrating the estimators on specific time series. As expected, the seasonal error of the Subscriptions and Donations Category is the lowest followed by the Insurance expenses. On the other hand, the Uncategorized expenses yield the highest seasonal error, denoting that there is low seasonal factor in this type of expenses.

The presented results highlight that the proposed methodology produces outcomes that can hardly accomplish the required approach based on the Coverage. In a probabilistic way, that is translated that for a given confidence level of e.g. 80%, the model is underperforming given the average values of the categories.

Furthermore, in Table 7 the performance of the Cash Flow prediction model applied to the enhanced analogous data is presented in terms of the same evaluation metrics. By comparing results between Tables 6 and 7, the main outcomes are that the seasonal error is lower in all categories. That can be explained as the analogous data preserves the seasonality factor since they are based on Fourier transform, highlighting the seasonal influence. This argument is also supported by the fact that, in terms of MAPE and MSE, the results are not better, which means that the mean value of the predicted distribution is not converge against the actual future values. Finally, we can express guarded optimism for the usage of analogous data as an enrichment technique, to be further explored since, in terms of coverage, this approach yields promising results, optimising the results of 14 out of 17 categories when 80% coverage is considered, while in 95% coverage 10 out of 17 illustrate better results.

**Table 7:** Average results per transaction category based on various metrics

Category (expenses)	Seasonal error	MASE	sMAPE	MSIS	Quantile Loss [0.5]	Coverage [0.5]	Quantile Loss [0.8]	Coverage [0.8]	Quantile Loss [0.95]	Coverage [0.95]
Assets	373.534	1.343	1.980	42.724	2811.85	0.466	3896.822	0.815	3899.518	0.915
Banking	640.346	69.007	1.949	2511.932	3172.123	0.47	4609.697	0.847	5036.039	0.924
Cash	3783.599	1.037	1.752	31.832	24129.639	0.452	32013.991	0.737	29854.857	0.862
Insurance	129.248	1.53	1.971	45.973	1165.007	0.464	1511.745	0.799	1477.439	0.908
Motor Vehicles Running Cost	527.229	2.873	1.841	62.53	4402.294	0.428	6080.678	0.737	5910.548	0.855
Offices	204.590	2.636	1.906	68.333	2635.013	0.457	3563.313	0.792	3514.37	0.881
Payroll & Benefits in Kind	342.144	2.471	1.9811	67.818	3503.126	0.474	5015.94	0.822	5383.268	0.917
Professional Fees	1589.156	13.40	1.887	475.538	15838.086	0.479	21370.288	0.789	19950.472	0.882
Rent & Lease	753.627	1.344	1.987	27.806	4977.029	0.474	7129.604	0.789	7523.01	0.900
Repairs & Maintenance	168.585	2.004	1.978	28.57	1541.248	0.468	2107.157	0.814	2150.599	0.925
Selling and distribution	637.919	5.337	1.783	163.050	5665.68	0.450	7665.938	0.757	7299.181	0.86
Subscriptions Donations	73.122	10.053	1.962	135.738	809.495	0.470	989.008	0.816	917.352	0.915
Supplier	1641.695	2.608	1.925	70.391	25259.910	0.455	31190.551	0.779	26794.230	0.882
Taxes	3139.324	0.431	1.955	12.492	16307.595	0.415	23018.600	0.662	22284.277	0.742
Transfer	7650.145	6.087	1.697	224.617	66887.980	0.442	78911.379	0.715	64099.914	0.842
Uncategorised	10193.153	3.110	1.759	97.321	84007.558	0.436	95502.407	0.714	72415.80	0.845
Utilities	243.400	4.33	1.923	116.887	2001.960	0.465	2730.252	0.788	2622.102	0.880

Surrogate Data used as an approach of dataset enrichment

**Fig. 12:**
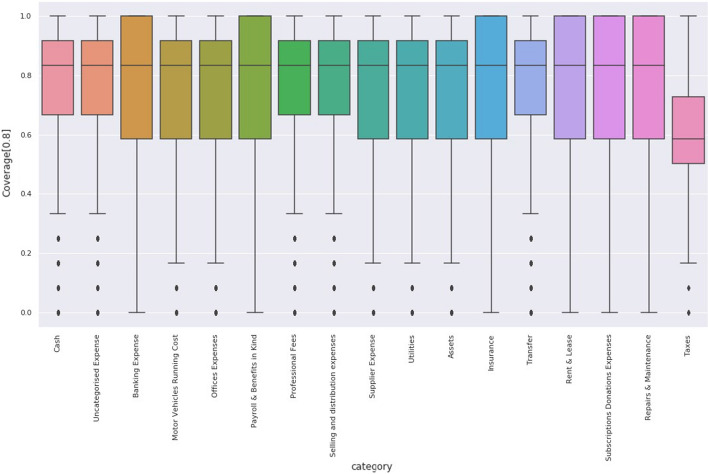
Boxplot of the 0.8 coverage, most of the categories have high variance while only the average of the taxes category seem to underperform

In Fig. 12 the same metric is depicted in a Bar Chart and we can conclude that the median of each category falls in the required threshold. The main reasoning of a poor evaluation performance is based on the long future of predictions (i.e. 12 weeks). Multi-step ahead prediction consists of predicting the next values of the time series and is performed in two different ways. The first one, independent value prediction, consists of training a direct model to predict the exact steps ahead value. The second, named iterative method, consists of repeating one-step ahead predictions at the desired horizon.

The iterative prediction only uses one model to forecast all the horizons needed. The objective is to analyze a short sequence of data while trying to predict the rest of the data sequence until a predefined time step is reached. The main drawback of this approach is the accumulative nature of the error. The implementation of a DeepAR model in our approach falls in this latter case, yielding higher error values when the prediction horizon widens.

## Conclusion

This paper sets the foundation for the development and provision of new banking services, tailor-made for SMEs, on top of the bank’s core activities. This is achieved by introducing a hybrid transaction classification model, which is in turn used for the development of an accurate Cash Flow prediction model based on DeepAR probabilistic forecasting model. Moreover, the classification task is extended towards the explainable AI aspect of the transaction categorization which assist interpreting the proposed model results while increasing transparency and trustworthiness. Having developed an accurate hybrid classification model, based on 20 Master Categories tailor-made for SMEs, we pave the way for the development of further banking microservices based on the results of the classification. Combined with the developed Cash Flow prediction model, those services could include:

1. Dynamic budget monitoring, where the DeepAR model can be extended to predefined set budgets per category.
2. Fraud detection and transaction guard, where new transactions beyond a given confidence level set per category could indicate malicious actions and fraudulent attempts.
3. Sector analysis and benchmarking, by utilizing open-source data based on the available SME NACE code and comparative analytics, based on mutual business sector SME customers of the bank.
4. Extended prescriptive analytics services, focusing on the provision of business insights and added value in the SMEs functional areas or improved VAT reconciliation.

From the bank’s perspective, the proposed services will allow the bank to get a deeper understanding of their SMEs customers, enabling the provision of tailor-made personalized financial products, while also attracting new business customers, seeking to utilize the bank’s extended business financial management services as a cost-effective solution.

## Data Availability

The materials used within the context of this research is open-sourced while the utilized dataset is proprietary and can not be disclosed.

## Code availability

A Git repository containing all the code for this project will be shared upon acceptance.
